# Students as anatomy near-peer teachers: a double-edged sword for an ancient skill

**DOI:** 10.1186/s12909-017-0996-y

**Published:** 2017-09-08

**Authors:** Nomy Dickman, Alon Barash, Shmuel Reis, David Karasik

**Affiliations:** 1 Unit for Assessment and Evaluation, Faculty of Medicine in the Galilee, Bar-Ilan University, H. Szold St. 14, POB 1589, 1311502 Safed, Israel; 2 Anatomy Program, Faculty of Medicine in the Galilee, Bar-Ilan University, H. Szold St. 14, POB 1589, 1311502 Safed, Israel; 3 The Center for Medical Education, Faculty of Medicine in the Galilee, Bar-Ilan University, Safed, Israel; 4 Current address: The Hebrew University-Hadassah School of Medicine, Jerusalem, Israel; 5 Faculty of Medicine Hebrew University, P.O.B 12272, 9112102 Jerusalem, Israel

**Keywords:** Undergraduate medical education, Near-peer teaching, Student instructors, Preclinical courses

## Abstract

**Background:**

A near-peer instructors (NPI) program was designed for 1st year medical students who successfully finished the Anatomy course, in order to develop their didactic ability and teaching skills, mostly for cadaver dissection.

**Methods:**

Graduates of the training program were administered a voluntary survey at the end of the program, annually. Best graduates of the training program were offered a NPI position in the next academic year. They were evaluated by the first-year students, at the end of the Anatomy block.

**Results:**

In a debriefing questionnaire at the end of the NPI training, on the five-point Likert scale (1 = lowest to 5 = highest), the overall rating ranged from 3.63 in 2013 to 3.71 in 2015. Learning prosection and anatomy demonstration skills scored on average from 4.30 to 4.36, respectively. The NPIs were then evaluated by first-year students at the end of the next year’s Anatomy block. On the Likert scale, the average score of NPIs ranged from 4.10 in 2014 to 4.75 in 2016, on the par with the general satisfaction score for the professional preclinical teachers during the same period (which ranged from 3.80 to 4.26).

**Conclusions:**

It is suggested that students as near-peer instructors can make a valuable contribution to the teaching faculty, especially in a new medical school.

## Practice points

- Near-peer teaching has demonstrable benefits for both sophomore tutors and their freshman students
- There appears to be no difference in the satisfaction of anatomy instruction provided by more senior peers compared to the faculty members.
- Providing didactic series on education to the top students of the anatomy class pay dividends both to the school and the students
- Teaching peers at an undergraduate level may be an exciting method for fostering a medical educator pathway

## Background

In response to the shortage of medical doctors in Israel [12, 17], a new Faculty of Medicine was established by Bar-Ilan University in 2011, at its new campus in the city of Safed, the Galilee region, Northern Israel. It is a four-year program, similar in its prerequisites to United States-based medical school programs. The Human Gross Anatomy course is allocated 240 h, set up as an 8.5-weeks block, during which ca. 70 first-year students learn about and dissect the entire body, excluding the brain, which is studied in a separate Soul-and-Mind block. Aside from lectures and dissections, hands-on radiology and ultrasound imaging are also incorporated into the curriculum using a dissection manual prepared by the anatomy faculty (DK and AB).

Basic science and clinical teachers were recruited overseas and locally, including physicians from hospitals affiliated with the faculty. However, junior faculty, such as anatomy laboratory teaching assistants (TAs), were difficult to find, as knowledgeable anatomy instructors based on graduate students’ pool are not readily available in this locale. The need to overcome this shortage of qualified anatomy dissection instructors was a driving force to initiate a near-peer teaching program to train such instructors for acquiring anatomic knowledge, skills and didactic ability, to be applied in the next academic year.

Near-peer teaching is an educational platform, whereby students are taught either by more senior students or their own classmates, which draws on their similar knowledge base and shared generational experiences [8]. It has been used previously in medical education training [1] for teaching gross anatomy, clinical skills and other content areas in the US and internationally, including the United Kingdom [8], Mexico [5] and China [3].

The literature indicates that students as peer teachers can make a great contribution to the teaching faculty [5]. A near-peer teaching program was developed for student volunteers in the Galilee Medical School. The goal of this report is to describe the design and implementation of near-peer teaching as applied to the anatomy curriculum and to share students’ evaluations of initial three years of experience.

## Methods

The Near-Peer Instructors (NPIs) program in the Galilee Medical School, is a series of eight sessions spread over the end of the second semester or start of the summer break in each academic year. The program was set up by the Department of Medical Education and the Anatomy Program. It has two main objectives: (1) to offer additional exposure in advanced anatomy to interested students beyond other preclinical classes during their first year, (2) to prepare near-peer instructors to assist the teaching faculty in the after–class hours dissections offered to the first-year Anatomy class.

Twelve first-year preclinical students attended the “elective” training program in 2013, 14 in 2014, and 15 in 2015. The participation in this training was voluntary, although we only invited students who graduated their anatomy class with a grade of 85 (out of 100) or above. Program’s content included the basics of didactics and pedagogic approaches and teaching methods, i.e. constructivist learning theory [7] and active learning - in the framework of andragogy (adult learning) - in large and in small group setting [9]. Specific modules included the use of active learning and technology in a large group setting, facilitation skills in a small-group setting, writing reflections [2, 4] and methods for evaluation of the students with an emphasis on providing effective feedback [20], as well as advanced practical skills in cadaveric dissection (similar to [11]). Hands-on sessions in ultrasound and guest lectures by specialists were also parts of the program. The program was based on a principle of cooperative learning, with reciprocal peer teaching, where students alternate roles as teacher and student [10]. Providing ongoing verbal and written peer evaluations was a requirement for the participants; each session was evaluated by participant feedback rubrics developed by the students under supervision [13, 15, 18]. The students presented a case-based learning (CBL) session or a mini-lecture on anatomical topic of their choice, and performed a prosection, with their performance being evaluated by the peers using a questionnaire (similar to [19]).

The discussions included the ethical values of cadaver donation and dissection, since the NPIs often face such questions from their more junior peers. A voluntary survey was administered at the end of the program. It included a debriefing questionnaire aimed at assessing both the general satisfaction with the program and the teachers, and the perceived value of the near-peer teaching experience.

Best graduates of the “elective” training program (up to 6 a year, selected based on their peer evaluations) were offered a NPI position during first semester of the next academic year. They were charged to manage afternoon sessions in the dissection room (with optional participation of the freshmen). They were evaluated by the first-year students, at the end of the Anatomy block, similar to the professional teachers and instructors (using the Likert five rank scale, with 1 = lowest to 5 = highest). Differences in the average grades were assessed by t-test, after testing the normality of their distribution.

## Results

**Fig. 1:**
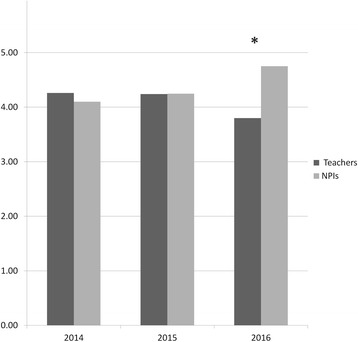
Student assessment of the professional teachers and near-peer instructors, by year. Asterisk – t-test *p*-value <0.0001

Here are the observations from the training program and NPI involvement into the Anatomy block:

- The NPI training program
  A debriefing questionnaire was administered at the end of the course, comprising of quantitative questions and free-language comment fields. On the Likert five rank scale (1–5) the overall rating for the “elective training course” was 3.85 ± 0.93 in 2013, 3.63 ± 0.52 in 2014, and 3.71 ± 0.73 in 2015 (Table 1). Learning prosection and anatomy demonstration skills scored 4.30 and 4.33 in 2013 and 2014, and 4.36 in 2015. Although the students agreed the CBL and prosection preparation experience increased their understanding of the topics they taught, the score for whether it improved their teaching style/skills was lower, at 3.70–3.75 in 2013, 3.33–3.56 in 2014, and 3.14 to 3.50 in 2015.
  In the qualitative comments the participants appeared at ease in the dissecting room amongst their peers. The participants thought they were able to prepare and lead a CBL session with confidence. Some even expressed their surprise in finding out that their classmates are good teachers who can offer a lot. The students pointed out that the course helped them to develop essential teaching skills and universal competencies, which would support their professional growth throughout the medical school. Among their suggestions for the future course, adding more of a prosection/dissection exposure and other hands-on exercises (such as ultrasound) was the most asked for.
- NPIs evaluation during the Anatomy block
  Best graduates of the “elective” training program were then offered a NPI position during the first semester of the next academic year (in their sophomore year). They were charged with instructing the first-year students during the dissection of the entire body in an apprenticeship model (usually with a seasoned anatomist in close proximity), in small-group afternoon sessions. Due to financial restraints, we were able to employ only the top training program graduates (four to 6 annually, selected based on their peer evaluations). NPIs were evaluated by their more junior peers at the end of the Human Anatomy 8.5-week-long didactic block. In 2014, of 70 students, 41 feedback forms were returned (~58%), the average rating for the overall quality of the sessions was 4.3 out of 5.0 (on a Likert 1 to 5 scale). For the professional teachers (2 preclinical faculty: A.B. and D.K., 3 affiliated physicians, and a physiotherapist), the average satisfaction score was 4.26 ± 0.69. For each of the four NPIs, 32 to 39 feedback forms were available; the average score was 4.10 ± 0.88, somewhat lower (non-significant) than the general satisfaction score for the professional preclinical teachers. In 2015, both NPIs and the professional teachers received almost identical scores (4.24 ± 0.69 and 4.25 ± 0.99), and in the 2016, the NPIs received significantly higher score than the professional teachers, 4.75 ± 0.44 and 3.80 ± 0.73, respectively (*p*-value <0.0001). Figure 1 illustrates average scores for both groups.
  The most common qualitative feedback received from the freshmen in regard to sophomore students was related to their ability of explaining the material well and expressions of gratitude.

**Table 1:** Student opinions about the NPI program

“to what extent are you satisfied/agree with the following”:	2013 average	±S.D.	2014 average	±S.D.	2015 average	±S.D.
- practice in the dissection room	4.30	0.57	4.33	0.87	4.36	0.84
- the course augmented my ability to teach my colleagues	3.75	1.02	3.33	0.71	3.50	0.85
- the course augmented my teaching style	3.70	0.92	3.56	1.01	3.14	0.95
General satisfaction from the course and its components	3.85	0.93	3.63	0.52	3.71	0.73
Number of responders		12		14		15

## Discussion

The evidence gathered after attending the “elective” NPI training program suggests that students gained confidence as anatomy instructors. Developing rubrics with students’ participation enabled them to be more self-directed and self-sufficient. The benefits to both the Faculty of Medicine and student instructors themselves are summarized below.

Our results concord with Bulte’s et al. suggestion (2007) that since teaching is an important skill for both future residency training and practice, training for teacher’s role should be introduced early on, i.e. in medical school [1]. We support a need to overcome a drawback noted by Lachman et al. [11], who pointed out that “while most medical students are enthusiastic about their future role as resident-educators, both students and residents feel uncomfortable teaching their peers due to the lack of necessary skills” [11]. The program’s participants recognized that the experience indeed equipped them with more advanced teaching skills that will be required as they move forward [6]. Students agreed that near-peer teaching helps prepare them for their future roles as health care professionals.

Notably, the sessions with hands-on prosection and anatomy demonstration skills scored highest – from 4.30 to 4.36, which attests to an interest the students have in continuous anatomy. Careful prosections by the training course participants are an important learning and teaching tool and a skill for future surgeons, although it is too early to predict whether graduates of NPI “elective” will choose surgical fields. Notably, these skills allow the maximization of the use of increasingly scarce gross anatomic resources (cadavers) by trained student instructors.

One interpretation of the difference between the prosection and anatomy demonstration skills and overall rating for the training course (from 3.63 to 3.85) and the self-evaluation of “improvement of teaching skills” (from 3.33 to 3.75) probably resides in the student perception that gaining anatomical skills was easier than gaining didactic/communication skills and feedback proficiency. Development of communication skills is also very valuable, although this NPI program did not explicitly teach these skills. Nevertheless, the “elective” participants agreed the CBL, small-group lecture and prosection preparation experience increased their understanding of the topics they taught. Certainly, improving NPIs communication and pedagogic skills can be applied beyond anatomy, both while still in medical school and to their careers as future physicians [10].

The advantage for the near-peer teachers is the opportunity to reinforce and expand their own learning style, develop essential teaching skills [6] and universal competencies, which would support their professional growth throughout the school. By assuming the responsibility of teaching their peers, students not only improve their understanding of course content, but also develop communication, team work and leadership skills, confidence and respect for peers that are vital to developing professionalism early in their medical careers [10]. Teaching others triggers the active learning process and the self-discovery [14].

The NPI program has also provided the school with an additional valuable and appropriate resource for teaching anatomy to first-year students, who themselves view the inclusion of near-peer teachers as a positive element in their learning (similar to [6]). Near-peer teaching is a viable option that satisfies the demands of modern curricula by using small groups. This format stimulates learning within courses that have large numbers of students and low faculty-to-student ratios [5]. Therefore, it is a win-win approach for both academic institution and the student’s body. In comparison with the seasoned professional teachers, whose average scores ranged from 3.80 to 4.26, the NPI’s scores were even higher, from 4.10 to 4.75. Similarly to our findings, help by the NPIs during dissection and prosection rated among the most valued experiences by 34.9% of the freshmen [5]. Generally, peer teaching is beneficial to both students and faculty [14] and compares favorably to faculty teaching [16].

Among the lessons learned for the future are: a need to provide more of a radiological hands-on experience (such as use of ultrasonography) and forays into the anatomy of childhood. The next elective program is scheduled to start in early 2017; it is planned to span over 40 h, to include these suggestions.

Among the potential limitations of this study is the representativeness of the course participants, as it consists of a modest self-selected sample. Evaluations of the near-pear instructors by the freshmen are presently available for the three consecutive course graduates only, based only on the feedback from course participants and the students whom they co-taught. There is therefore a concern of how generalizable the above results are to all pre-clinical students. This study was not intended to estimate whether the near-peer teaching increased student’s retention of information they taught to their peers or influenced their professional career path.

## Conclusions

In conclusion, both sophomore NPIs and first-year students enjoyed the experience and near-peer co-operation. The results from this teaching development support the use of a near-peer instructor as a small-group facilitator, especially during the human gross anatomy dissection. Given the potential positive educational value of near-peer teaching, it seems to be appropriate to be used at a larger scale to enhance the learning experience. Future study should address the long-term effects on students’ confidence in applying this learning tool to other pre-clinical fields. While this study focused on anatomical education, the authors believe that the use of near-peer teaching is an important component of medical school curriculum and contributes to developing teaching skills in a flourishing community of learning.

## Abbreviations

NPIs: Near-Peer Instructors
TAs: Teaching Assistants
